# Optimizing pureed diets via texture analysis: A study on the impact of different energy levels and ingredient ratios on nasogastric tube patency

**DOI:** 10.1371/journal.pone.0329207

**Published:** 2025-08-29

**Authors:** Muxi Chen, Dongyu Mu, Yi Cheng, Lingli Zhang, Lei Shi, Yuan Liu

**Affiliations:** 1 Department of Clinical Nutrition, West China Hospital, Sichuan University, Chengdu, China; 2 Clinical Medicine College, Sichuan College of Traditional Chinese Medicine, Mianyang, China; 3 Department of Pharmacy/Evidence-Based Pharmacy Center, West China Second University Hospital, Sichuan University, Chengdu, China; 4 Children’s Medicine Key Laboratory of Sichuan Province, Chengdu, China; Yantai Institute of Technology, CHINA

## Abstract

**Objective:**

This study aimed to investigate the impact of different energy levels and ingredient ratios on the nasogastric tube patency of pureed diets, optimizing the formulations to meet the nutritional requirements of elderly nasogastric feeding patients while minimizing tube blockage risk.

**Methods:**

The study followed the guidelines of the “*Chinese Resident’s Balanced Diet Pyramid*” and formulated five different energy levels of pureed diets (900 kcal, 1200 kcal, 1500 kcal, 1800 kcal, and 2100 kcal) using natural food groups. The diets consisted of seven major food categories: cereals and tubers, vegetables, meats, milk, oil, salt, and fruits. The liquid formulations for the above energy levels were prepared according to the concentration standards for special medical purpose foods (FSMPs). The maximum injection force required for nasogastric feeding was measured via a texture analyzer. The nutritional components of the pureed diets at different energy levels and ingredient ratios were evaluated via West China Hospital Nutrition Software. Spearman correlation analysis, multiple regression analysis, and random forest models were used to explore the relationships between energy levels, nutritional components, ingredients, maximum injection force, and tube patency.

**Results:**

The study revealed that as the energy density increased, the maximum injection force of the pureed diets significantly increased (*p* < 0.05), particularly at the 2100 kcal energy level, where the “rice‒carrot‒beef” formula reached the highest value (117.59 ± 0.26 N), whereas the “FSMP” formula at 900 kcal presented the lowest injection force (9.62 ± 0.20 N). There was a significant difference in the impact of different energy levels and formulations on the maximum injection force (*p* < 0.05). Spearman correlation analysis indicated that carbohydrate (*ρ* = 0.736) and dietary fiber (*ρ* = 0.668) contents were significantly positively correlated with the maximum injection force (*p* < 0.05). Multiple regression analysis further revealed that carbohydrates were the primary factor influencing the injection force, with a regression coefficient of 0.247 (*p* < 0.05), suggesting that each additional gram of carbohydrate increased the maximum injection force by approximately 0.247 N, whereas the effects of protein, fat, and dietary fiber were not significant (*p* > 0.05). All nutritional components (energy (*ρ* = 0.629), carbohydrates (*ρ* = 0.621), protein (*ρ* = 0.582), fat (*ρ* = 0.547), and dietary fiber (*ρ* = 0.544)) were significantly positively correlated with tube blockage (*p* < 0.05). Mann‒Whitney U tests revealed that the energy, carbohydrate, protein, fat, and dietary fiber contents in the tube blockage group were significantly greater than those in the nonblockage group (*p* < 0.05). With respect to food categories, cereals (*ρ* = 0.742) and meats (*ρ* = 0.766) were significantly positively correlated with the maximum injection force (*p* < 0.05). Specifically, rice (*ρ* = 0.7886) and sweet potato (*ρ* = 0.506) were significantly positively correlated (*p* < 0.05), whereas rice flour (*ρ* = −0.411) and milk (*ρ* = −0.690) were significantly negatively correlated (P < 0.05). Moreover, cereals (*ρ* = 0.615) and meats (*ρ* = 0.628) were significantly positively correlated with the risk of tube blockage at all energy levels (*p* < 0.05), with rice (*ρ* = 0.660) and beef (*ρ* = 0.153) significantly increasing the risk of blockage, whereas rice flour (*ρ* = −0.350) and milk (*ρ* = −0.557) were significantly negatively correlated with the risk of blockage (P < 0.05). The random forest model’s feature importance analysis revealed that carbohydrates (33.33%) and dietary fiber (23.01%) were the most important factors for predicting tube blockage, with an AUC value of 0.91, indicating strong predictive ability.

**Conclusion:**

This study explores the impact of nutritional components and ingredient characteristics on tube patency and blockage risk in nasogastric pureed diets, revealing key optimization pathways for pureed diet formulations. The energy density and ingredient selection of pureed diets significantly affect tube patency. High-energy diets provide higher nutritional density but significantly increase the injection force and blockage risk. Diet formulations should be optimized by reducing high-viscosity and high-hardness ingredients such as rice and beef, using rice flour to replace rice, and milk as the liquid component. For high-energy demands, the carbohydrate and dietary fiber contents should be controlled to reduce the injection force requirements and blockage risk. The study also developed a five-dimensional blockage risk warning model based on energy, protein, fat, carbohydrate, and dietary fiber (AUC = 0.91), classifying low-, medium-, and high-risk levels. Low-risk patients (energy≤1400 kcal/d, carbohydrates≤200 g/d, protein≤70 g/d) are recommended to use homemade formulas, whereas high-risk patients (energy≥1601 kcal/d, carbohydrates≥241 g/d, protein≥86 g/d) should use FSMP for full feeding to balance nutritional supply and tube patency. The findings of this study provide both theoretical and practical guidance for optimizing diets for dysphagia patients, emphasizing that adjusting formulations can effectively balance nutritional supply and tube patency, reduce blockage risk, and prevent malnutrition in homemade pureed feed. This has significant implications for reducing nasogastric complications and ensuring the safety of medical procedures.

## 1. Introduction

As of 2023, the population of elderly individuals aged 60 years and above in China has reached 297 million, accounting for 21.1% of the total population [1]. With the acceleration of population aging, the demand for healthcare among the elderly has been increasing annually. Among these, the number of elderly patients requiring nasogastric tube feeding is also increasing, making nutritional support for elderly patients a key topic in clinical care. According to statistical data, 12.7% of the elderly population aged 65 years and above in China requires long-term nutritional support, with more than 40% of these cases related to swallowing dysfunction (FOIS grade 3 or less) requiring nasogastric feeding [2]. Investigations have revealed that 6% of elderly individuals aged 65 years and above in a nursing institution in Tianjin rely on nasogastric nutritional support, with this proportion increasing to 20.8% among hospitalized neurology patients [3].

Nasogastric tube feeding, as a critical intervention for ensuring patient feeding safety and maintaining nutritional intake, has established clear operational standards in clinical practice. Although this nutritional intervention effectively mitigates the risk of aspiration and meets basic metabolic needs, there are deep conflicts between cultural traditions and medical regulations in actual clinical settings [4]. Notably, care for nasogastric tube patients in China has significant local characteristics. Despite the “Enteral Nutrition Clinical Guidelines” recommending standardized nutritional formulations, 68.3% of family caregivers opt for homemade pureed diets in practice. This tendency is rooted in the traditional belief that “food as medicine” — surveys show that 92.6% of caregivers believe that “natural food has better nutritional affinity,” 77% of consumers are convinced that natural ingredients provide a more complete nutritional profile, and 81.3% of families choose homemade pureed diets on the basis of the belief that “traditional diets are more in line with the patient’s taste memory” [5]. Market research further revealed that the sales growth rate of enteral nutrition products containing natural ingredients has reached 22.4%, which is significantly higher than the 8.7% growth rate of synthetic products [6].

However, it is important to note that there is currently a lack of systematic nutritional guidance for the preparation of pureed diets in clinical settings, including key parameters such as ingredient ratios, energy density control, and nutrient retention rates, all of which exhibit significant individual variability. This experience-driven model of nutritional supply may lead to a discrepancy between the actual nutrient intake of patients and their clinical needs, potentially affecting disease progression and quality of life. Therefore, establishing a scientifically regulated pureed diet application system has become an important issue for improving the quality of medical care for elderly nasogastric feeding patients.

## 2. Methods

### 2.1. Selection of research subjects

The pureed diet formulations in this study included cereals and tubers, vegetables, meats, eggs, milk, canola oil, salt, and fruits. In accordance with the 2024 “*Chinese Food Composition Table*” [7], the quartile method was used to identify representative ingredients. Three types of vegetables (carrots, Chinese cabbage, and celery), three types of meats (pork, beef (hind leg), and basa fish), and two types of cereals and tubers (rice and sweet potato) were selected. Additionally, rice flour, which is produced from rice through grinding and dehydration processes, is included as a cereal ingredient.

The specific selection criteria are as follows:

(1) Vegetables: On the basis of their dietary fiber content, typical vegetables with representative fiber contents at the quartile points were selected: carrots (dietary fiber content: 1.1 g/100 g), Chinese cabbage (dietary fiber content: 1.5 g/100 g), and celery (dietary fiber content: 2.2 g/100 g).
(2) Cereals and tubers: The following representative cereals and tubers were chosen on the basis of dietary fiber content: rice (dietary fiber content: 0.5 g/100 g), sweet potato (dietary fiber content: 1 g/100 g), and glutinous rice (dietary fiber content: 2.8 g/100 g). Owing to the high viscosity of glutinous rice, which makes it difficult for patients to consume it, glutinous rice was not selected as an experimental ingredient.
(3) Meats: Three representative meats were selected on the basis of fat content: beef (hind leg) (fat content: 2 g/100 g), basa fish (fat content: 4.1 g/100 g), and pork (fat content: 13.8 g/100 g).
(4) Fruits: Apple was chosen as the representative fruit, based on the recommended daily fruit intake in the Chinese Dietary Guidelines and the Chinese Food Pyramid [8]. It was selected for its accessibility and its ability to provide sufficient dietary fiber and vitamins.

### 2.2. Preparation of pure diets

The pureed diet formulations in this study were based on the recommended intake proportions from the “*Chinese Resident’s Balanced Diet Pyramid*” [8]. Five different pureed diets were designed on the basis of varying energy levels: 900 kcal, 1200 kcal, 1500 kcal, 1800 kcal, and 2100 kcal.

The specific formulations are as follows:

(1) Formulation with tubers (energy levels: 900 kcal, 1200 kcal, 1500 kcal, 1800 kcal, and 2100 kcal)

**Table:** 

Energy (kcal)	Rice (g)	Sweet Potato (g)	Vegetables (g)	Meats (g)	Egg (g)	Milk (g)	Canola Oil (g)	Salt (g)	Fruits (g)
900	85	50	200	35	50	500	10	1	150
1200	125	75	250	75	50	500	15	2	150
1500	175	100	300	125	50	300	25	4	200
1800	225	125	400	175	50	300	25	4	200
2100	275	150	450	225	50	300	25	4	300

(2) Formulation without tubers (energy levels: 900 kcal, 1200 kcal, 1500 kcal, 1800 kcal, and 2100 kcal)

**Table:** 

Energy (kca)	Rice/Rice Flour (g)	Vegetables (g)	Meats (g)	Egg (g)	Milk (g)	Canola Oil (g)	Salt (g)	Fruits (g)
900	85	300	25	50	500	10	1	150
1200	125	450	75	50	500	15	2	150
1500	175	600	125	50	300	25	4	200
1800	250	750	175	50	300	25	4	200
2100	275	900	225	50	300	25	4	300

The preparation process for the pureed diets included the following steps:

(1) Ingredient processing:

Cereals: Rice was steamed at a ratio of rice to water (1:2) for 30 minutes. Rice flour was mixed with water at a 1:2 ratio to form a rice paste. The sweet potato was cleaned, cut into pieces, and steamed for 30 minutes.

Vegetables: Carrots, Chinese cabbage, and celery were cleaned, cut into pieces, and steamed for 30 minutes.

Meat: Pork, beef (hind leg), and basa fish are deboned, fat is removed, and the meat is cut into pieces and steamed for 30 minutes.

Fruits: The apple was washed, peeled, cut into pieces, and then blended and mixed to achieve the desired consistency.

Other ingredients: Eggs are boiled, milk is added according to the specified proportion, and canola oil and salt are used for blending.

(2) Blending: After processing, all ingredients are added in predetermined proportions to a blender for separation, which is performed for 5–8 minutes. Importantly, no additional water is added other than the natural moisture from the ingredients themselves.

### 2.3 Data collection

In total, the study included five energy levels (900, 1200, 1500, 1800, and 2100 kcal), each with 16 distinct formulations constructed from different combinations of cereals, vegetables, and meats, resulting in 80 unique pureed diet samples. For each formulation, the maximum injection force was measured in triplicate using the texture analyzer to ensure precision and repeatability. This yielded a total of 240 measurements for statistical analysis. These measurements form the complete dataset for evaluating injection force trends, nutrient correlations, and blockage status in the subsequent analysis.

#### 2.3.1 Measurement of the maximum injection force.

A texture analyzer was used to measure the maximum injection force required for different energy levels and formulations of pureed diets to pass through a 60 mL manually operated syringe (parameters: total length 18 cm, nozzle length 3.3 cm, nozzle inner diameter 5 mm, nozzle outer diameter 7 mm, barrel inner diameter 28 mm, barrel outer diameter 32 mm) connected to an F16 nasogastric tube (inner diameter: 5.3 mm). This measurement was used to assess tube patency. The texture analyzer recorded the injection force (N), which represents the resistance encountered by the pureed diets during nasogastric tube passage. Each formulation was tested three times to ensure data reliability and accuracy.

#### 2.3.2 Nutritional composition data.

The nutritional composition of the pureed diets at different energy levels (900, 1200, 1500, 1800, and 2100 kcal) was evaluated and calculated via professional nutritional analysis software from the “West China Hospital Nutrition System”, which features a comprehensive food database and high-precision nutritional calculation capabilities. This software performs detailed assessments on the basis of specific ingredient types, weights, and combination ratios to ensure scientific accuracy and precision in the analysis.

### 2.4 Determination of the blockage status

The alarm threshold for enteral nutrition pumps varies by model and manufacturer. For example, the SN-600 N(R) enteral nutrition pump produced by Shenzhen Shengnuo Medical Equipment Co., Ltd. has a blockage alarm threshold of 500 ± 200 mmHg, approximately equivalent to 66.7 ± 26.7 kPa. Typically, the alarm setting for enteral nutrition pumps is set at approximately 70 ± 30 kPa [9,10]. In this study, a 60 mL manually operated syringe was used, and when the pressure reached 70 ± 30 kPa, the corresponding force required was approximately 43.10 ± 18.47 N. Therefore, a threshold of 43.1 N was defined as the criterion for tube blockage in this study.

### 2.5 Data analysis

Descriptive statistical analysis and nonparametric statistical tests were used to evaluate the maximum injection force of pureed diets with different energy levels and formulations during nasogastric feeding and to examine their relationship with blockage status. All the statistical analyses were conducted via SPSS 25.0, with the significance level set at *p* < 0.05.

#### 2.5.1 Analysis of the injection force across different energy levels.

Descriptive statistics were used to calculate the mean and standard deviation of the maximum injection force for pureed diets at different energy levels (900, 1200, 1500, 1800, and 2100 kcal) to assess the overall trends and variation in the injection force. The Kruskal‒Wallis test was applied to determine significant differences in tube patency among different energy levels and formulations.

#### 2.5.2 Normality and homogeneity of variance testing.

To determine whether the injection force data followed a normal distribution, the Shapiro‒Wilk test was performed. The results yielded a test statistic of 0.912, *p* < 0.05, indicating a significant deviation from normality. Furthermore, the Levene test for homogeneity of variance showed a test statistic of 4.82, *p* < 0.05, indicating significant differences in variance among groups. Therefore, nonparametric statistical methods were employed to accommodate the data characteristics.

#### 2.5.3 Correlation analysis between nutritional composition, ingredients, and maximum injection force.

To examine the relationships between key nutritional components (energy, protein, fat, carbohydrates, and dietary fiber) and the maximum injection force, Spearman correlation analysis was conducted. Additionally, the associations between different food ingredients (e.g., cereals, tubers, meats, vegetables) and the maximum injection force during nasogastric feeding were analyzed to explore the impact of ingredient selection on tube patency.

#### 2.5.4 Correlation analysis between nutritional composition and blockage status.

To investigate the relationship between nutritional composition and tube blockage, Spearman correlation analysis was performed between blockage status (a binary variable: 1 = blocked, 0 = nonblocked) and key nutritional components (energy, protein, fat, carbohydrates, and dietary fiber). Furthermore, the Mann‒Whitney U test was applied to compare the nutritional composition between the blocked and nonblocked groups to identify significant differences.

#### 2.5.5 Regression analysis of the maximum injection force.

Linear regression analysis was used to explore the specific relationship between the energy density and maximum injection force. The regression model assessed whether increasing the energy density significantly influenced the maximum injection force. Additionally, multiple linear regression analysis was conducted to determine the independent contributions of different nutritional components to the injection force.

#### 2.5.6 Multivariate analysis of the impact of nutritional components on blockage status.

A logistic regression model and random forest model were used to quantify the independent effects of various nutritional components on tube blockage. These models assess the predictive power of different nutritional factors for tube blockage and rank their relative importance.

## 3 Results

### 3.1 Effects of different energy levels and formulations on the maximum injection force

**Fig 1:**
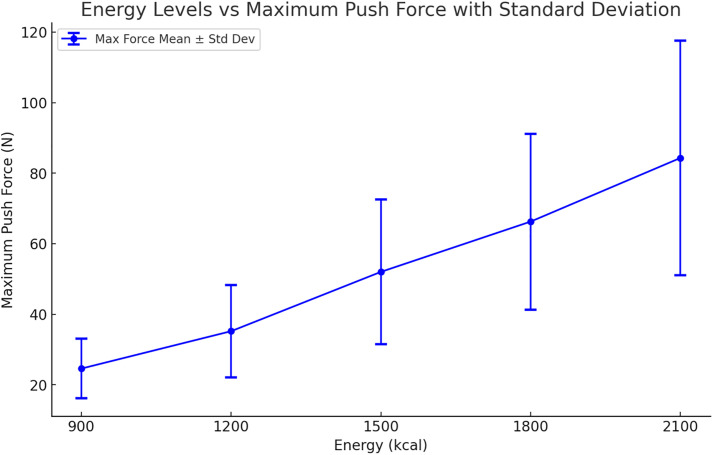
Trend of the maximum injection force across different energy levels.

Descriptive statistics (mean±standard deviation) were performed on the maximum injection force data for nasogastric feeding at different energy levels (900 kcal, 1200 kcal, 1500 kcal, 1800 kcal, and 2100 kcal) to assess the impact of varying energy density. The results indicated that as energy density increased, the maximum injection force also increased, with significant differences across the different energy levels (*p* < 0.05). The highest injection force was observed at the 2100 kcal level, particularly in the “rice-carrot-beef” formulation, which had the highest maximum injection force of all combinations (117.59 ± 0.26 N). In contrast, the “Li Heng Yun” (FSMP) formulation at 900 kcal had the lowest maximum injection force (9.62 ± 0.20 N) (Fig 1).

The Kruskal-Wallis test revealed significant differences within each energy level and across formulations (*p* < 0.05), indicating that energy density significantly influences the injection force required for different formulations. (Table 1).

**Table 1:** Maximum injection force of pureed diets at different energy levels and formulations (N) (mean ± standard deviation) and statistical significance*.

Formulation	Energy				
	900 kcal*	1200 kcal*	1500 kcal*	1800 kcal*	2100 kcal*
Li Heng Yun (FSMP)*	9.62 ± 0.20	14.57 ± 0.18	15.12 ± 0.14	18.94 ± 0.75	22.81 ± 0.70
Rice-Carrot-Pork*	16.43 ± 0.05	24.36 ± 0.37	38.72 ± 0.27	76.48 ± 0.60	97.46 ± 0.43
Rice Flour-Carrot-Pork*	15.43 ± 0.55	21.70 ± 0.13	32.89 ± 0.78	40.92 ± 0.78	46.77 ± 0.58
Rice-Sweet Potato-Carrot-Pork*	28.91 ± 1.38	35.46 ± 1.27	58.84 ± 0.89	76.34 ± 0.94	99.10 ± 1.60
Rice-Carrot-Beef*	34.81 ± 0.81	52.71 ± 0.24	67.84 ± 0.83	84.59 ± 0.18	117.59 ± 0.26
Rice Flour-Carrot-Beef*	16.96 ± 0.82	24.85 ± 1.13	32.67 ± 0.42	46.10 ± 0.28	69.64 ± 0.10
Rice-Sweet Potato-Carrot-Beef*	33.14 ± 0.57	48.33 ± 0.42	59.70 ± 0.77	74.58 ± 1.24	103.64 ± 1.78
Rice-Carrot-Fish*	33.06 ± 0.06	58.69 ± 0.61	76.32 ± 0.28	88.62 ± 0.58	118.69 ± 1.06
Rice Flour-Carrot-Fish*	15.77 ± 0.34	24.79 ± 0.54	34.92 ± 0.69	44.53 ± 0.50	57.80 ± 0.23
Rice-Sweet Potato-Carrot-Fish*	30.13 ± 2.61	43.74 ± 0.30	72.27 ± 0.43	87.60 ± 0.38	100.03 ± 1.90
Rice-Celery-Pork*	15.86 ± 0.04	28.95 ± 0.85	47.77 ± 1.56	54.38 ± 0.31	66.76 ± 0.46
Rice Flour-Celery-Pork*	18.26 ± 0.81	21.56 ± 0.55	33.21 ± 0.16	41.16 ± 0.47	47.77 ± 0.71
Rice-Sweet Potato-Celery-Pork*	34.09 ± 0.78	44.27 ± 1.11	75.67 ± 0.52	98.43 ± 1.31	121.72 ± 0.41
Rice-Celery-Beef*	38.56 ± 0.45	51.47 ± 0.45	66.73 ± 0.52	86.23 ± 0.64	103.04 ± 0.87
Rice Flour-Celery-Beef*	18.08 ± 0.78	28.65 ± 0.47	34.06 ± 0.41	43.24 ± 0.20	49.44 ± 0.39
Rice-Sweet Potato-Celery-Beef*	38.20 ± 0.71	45.35 ± 0.44	77.10 ± 2.05	99.78 ± 0.33	126.10 ± 0.73
Rice-Celery-Fish*	28.19 ± 0.44	41.73 ± 1.03	59.85 ± 0.58	87.43 ± 1.81	115.28 ± 2.82
Rice Flour-Celery-Fish*	18.41 ± 0.75	20.68 ± 0.20	31.04 ± 0.75	42.07 ± 0.26	47.10 ± 0.93
Rice-Sweet Potato-Celery-Fish*	35.00 ± 0.80	57.07 ± 0.38	73.22 ± 0.65	98.23 ± 0.81	119.60 ± 0.20
Rice‒Cabbage-Pork*	15.99 ± 0.37	20.69 ± 0.46	61.36 ± 0.46	70.38 ± 0.33	78.65 ± 0.30
Rice Flour-Cabbage-Pork*	16.88 ± 1.27	24.97 ± 0.46	32.75 ± 0.19	36.52 ± 0.30	41.42 ± 0.02
Rice-Sweet Potato-Cabbage-Pork*	24.68 ± 1.27	39.50 ± 0.65	49.38 ± 0.31	86.71 ± 1.08	124.49 ± 2.68
Rice‒Cabbage-Beef*	25.82 ± 0.37	31.04 ± 0.80	35.05 ± 0.46	38.57 ± 0.26	71.91 ± 0.14
Rice Flour-Cabbage-Beef*	21.66 ± 0.65	25.92 ± 0.09	30.85 ± 0.67	38.41 ± 0.37	43.42 ± 0.15
Rice-Sweet Potato-Cabbage-Beef*	35.49 ± 0.42	48.98 ± 0.58	94.34 ± 0.24	100.53 ± 0.56	134.85 ± 1.93
Rice‒Cabbage-Fish *	19.93 ± 0.72	25.59 ± 0.25	49.67 ± 0.39	66.70 ± 0.93	74.62 ± 0.27
Rice Flour-Cabbage-Fish*	18.71 ± 0.05	24.23 ± 0.81	29.43 ± 0.37	33.41 ± 0.43	40.38 ± 0.47
Rice-Sweet Potato-Cabbage-Fish*	30.92 ± 1.24	55.93 ± 0.67	85.82 ± 0.16	94.71 ± 1.45	120.44 ± 1.45

### 3.2. Correlation analysis between nutritional composition and maximum injection force

Spearman’s rank correlation coefficient was used to assess the correlation under a nonnormal distribution. The results revealed that all nutritional components (protein, fat, carbohydrate, and dietary fiber) presented significant positive correlations with the mean maximum injection force (*p* < 0.05), with the strongest correlation observed between carbohydrate content and the maximum injection force.

**Fig 2:**
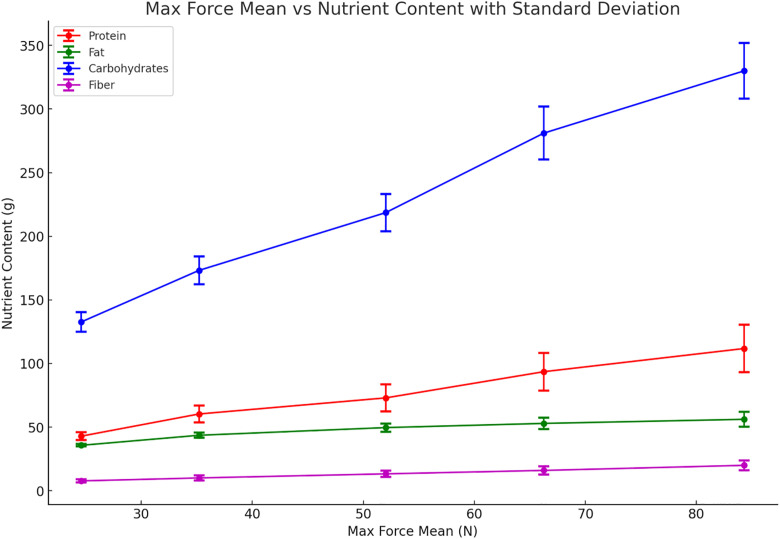
Relationships between the mean maximum injection force and nutritional components (including standard deviation).

Multiple regression analysis showed that carbohydrates were the only significant predictor of injection force (*β* = 0.247, *p *< 0.05), with each additional gram of carbohydrates increasing the injection force by approximately 0.247 N. In contrast, protein, fat, and dietary fiber were not significant predictors (Tables 2,3 and Fig 2).

**Table 2:** Spearman correlation analysis results. Between nutritional components and the maximum injection force.

Nutritional Component	Spearman Correlation Coefficient (*ρ*(rho))	*p* value
Protein (g)	0.708	<0.05
Fat (g)	0.662	<0.05
Carbohydrates (g)	0.736	<0.05
Dietary Fiber (g)	0.668	<0.05

**Table 3:** Multiple regression analysis results. Between nutritional components and the maximum injection force.

Variable	Regression Coefficient	Standard Error	t value	*p* value
Constant	−7.157	13.449	−0.532	>0.05
Protein (g)	0.186	0.151	1.231	>0.05
Fat (g)	−0.225	0.463	−0.485	>0.05
Carbohydrates (g)	0.247	0.0578	4.265	<0.05
Dietary Fiber (g)	0.115	0.755	0.152	>0.05

### 3.3. Correlation analysis between ingredients and the maximum injection force

#### 3.3.1. Spearman correlation analysis between ingredients and the maximum injection force.

Spearman correlation analysis was performed according to food category, revealing significant positive correlations between the quantities of cereals, tubers, vegetables, meats, oils, salts, and fruits and the maximum injection force (*p* < 0.05). Among the categories, cereals (*ρ* = 0.742) and meats (*ρ* = 0.766) presented relatively high correlation coefficients. A detailed analysis of specific ingredients revealed that rice (*ρ* = 0.7886) and sweet potato (*ρ* = 0.506) were significantly positively correlated (p < 0.05), whereas rice flour (ρ = −0.411) and milk (*ρ* = −0.690) were significantly negatively correlated (*p* < 0.05). However, the correlations between the maximum injection force and ingredients such as carrots (*ρ* = 0.094), cabbage (*ρ* = −0.073), celery (*ρ* = 0.103), pork (*ρ* = −0.016), beef (*ρ* = 0.169), and fish (*ρ* = 0.147) were not significant (*p* ≥ 0.05) (Tables 4 and 5).

**Table 4:** Spearman correlation analysis between ingredient categories and the maximum injection force.

Ingredient	Spearman Correlation Coefficient (*ρ*(rho))	*p* value
Cereals (g)	0.742	<0.05
Tubers (g)	0.506	<0.05
Vegetables (g)	0.314	<0.05
Meats (g)	0.766	<0.05
Milk (g)	−0.690	<0.05
Oil (g)	0.714	<0.05
Salt (g)	0.714	<0.05
Fruits (g)	0.722	<0.05

**Table 5:** Spearman correlation analysis between specific ingredients and the maximum injection force.

Specific Ingredient	Spearman Correlation Coefficient (*ρ*(rho))	*p* value
Rice (g)	0.7886	<0.05
Rice Flour (g)	−0.411	<0.05
Sweet Potato (g)	0.506	<0.05
Carrots (g)	0.094	≥0.05
Cabbage (g)	−0.073	≥0.05
Celery (g)	0.103	≥0.05
Pork (g)	−0.016	≥0.05
Beef (g)	0.169	≥0.05
Fish (g)	0.147	≥0.05
Milk (g)	−0.690	<0.05
Oil (g)	0.714	<0.05
Salt (g)	0.714	<0.05
Apple (g)	0.722	<0.05

#### 3.3.2. Kruskal‒Wallis test results for different ingredients and maximum injection forces.

**Fig 3:**
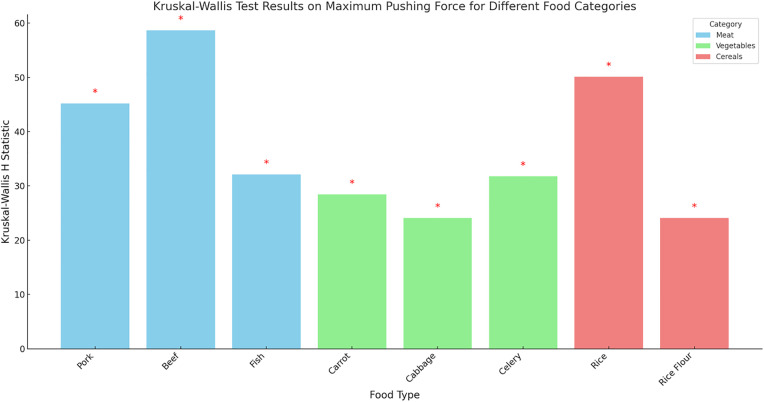
Kruskal‒Wallis test results and statistical significance for different means, vegetables, and cereals on the maximum injection force (Note: “*”: p ≤ 0.05).

The Kruskal‒Wallis test was performed for all food categories (cereals, tubers, vegetables, meats, milk, oils, salts, and fruits), and significant differences were detected (*p* < 0.05). Among these, cereals (H statistic = 83.070) and meats (H statistic = 80.128) exhibited the greatest differences. Further analysis of the impacts of different meats (pork, beef, and fish), vegetables (carrots, cabbage, and celery), and cereals (rice and rice flour) on the maximum injection force also revealed significant differences (*p* < 0.05). In the meat category, beef had the largest H statistic (58.67), followed by pork (45.21) and fish (32.14). In the vegetable category, celery had the largest H statistic (31.77), followed by carrots (28.45) and cabbage (24.11). Among cereals, rice had a larger H statistic (50.12) than did rice flour (24.11) (Table 6 and Fig 3).

**Table 6:** Kruskal‒Wallis test results for different ingredients and maximum injection forces.

Ingredient Category	Kruskal‒Wallis H Statistic	*p* value
Cereals (g)	83.070	<0.05
Tubers (g)	53.175	<0.05
Vegetables (g)	38.665	<0.05
Meats (g)	80.128	<0.05
Milk (g)	63.843	<0.05
Oil (g)	68.391	<0.05
Salt (g)	68.391	<0.05
Fruits (g)	70.620	<0.05

### 3.4. Correlation analysis between nutritional composition and blockage status

**Fig 4:**
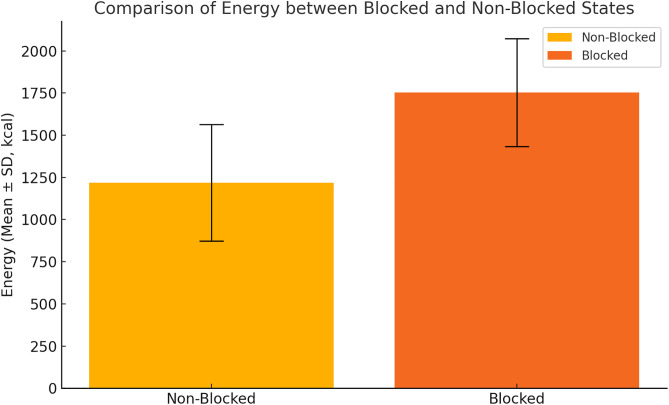
Comparison of energy intake between blocked and nonblocked statuses (mean ± standard deviation).

**Fig 5:**
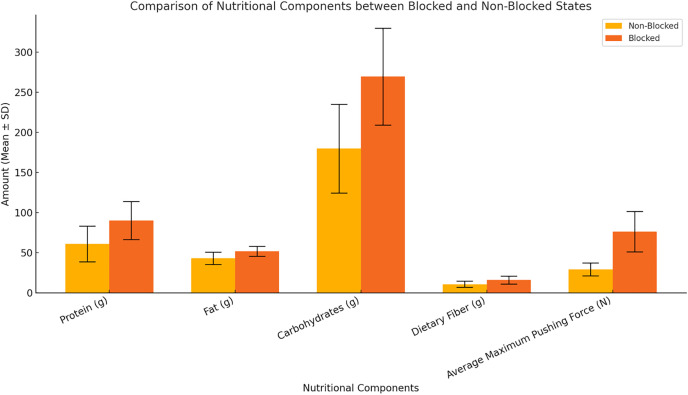
Comparison of nutritional components (protein, fat, carbohydrate, and dietary fiber) and the mean maximum injection force between the blocked and nonblocked states (mean ± standard deviation).

Spearman’s rank correlation and Mann-Whitney U tests showed that energy, protein, fat, carbohydrate, and dietary fiber were significantly higher in the blocked group compared to the nonblocked group (*p* < 0.05). The energy content in the blocked group was 1753.5 ± 319.30 kcal, while in the nonblocked group it was 1218.8 ± 345.89 kcal. Similarly, other nutrients such as protein, fat, carbohydrate, and dietary fiber showed significantly higher values in the blocked group (Figs 4 and 5).

Spearman correlation analysis revealed that all nutritional components (energy (ρ = 0.629), protein (ρ = 0.582), fat (ρ = 0.547), carbohydrates (ρ = 0.621), and dietary fiber (ρ = 0.544)) were significantly positively correlated with blockage status (*p* < 0.05). The Mann‒Whitney U test results revealed that the energy, protein, fat, carbohydrate, and dietary fiber contents in the blocked group were significantly greater than those in the nonblocked group (*p* < 0.05) (Tables 7 and 8).

**Table 7:** Spearman correlation analysis Nutritional components and blockage status.

Nutritional Component	Spearman Correlation Coefficient (ρ(rho))	*p* value
Energy (kcal)	0.629	<0.05
Protein (g)	0.582	<0.05
Fat (g)	0.547	<0.05
Carbohydrates (g)	0.621	<0.05
Dietary Fiber (g)	0.544	<0.05

**Table 8:** Nutritional Composition Differences between the “Blocked” and “Nonblocked” Groups (Mann‒Whitney U Test Results).

Nutritional Component	U Statistic	*p* value
Energy (kcal)	3892	<0.05
Protein (g)	3802	<0.05
Fat (g)	3708.5	<0.05
Carbohydrates (g)	3903	<0.05
Dietary Fiber (g)	3701.5	<0.05

### 3.5. Impact of different ingredients on blockage risk

Spearman’s correlation analysis revealed significant positive correlations between cereals, tubers, vegetables, meats, oils, salts, and fruits with blockage status (*p* < 0.05). Specifically, cereals (*ρ* = 0.615) and meats (*ρ* = 0.628) had the highest correlations. The correlation between ingredients and blockage risk varied, with rice and sweet potato positively correlating with blockage, while rice flour and milk showed negative correlations(*p *< 0.05).(Tables 9 and 10).

**Table 9:** Spearman correlation analysis between ingredient categories and blockage status.

Ingredient Category	Spearman Correlation Coefficient (ρ(rho))	*p* value
Cereals (g)	0.615	<0.05
Tubers (g)	0.399	<0.05
Vegetables (g)	0.260	<0.05
Meats (g)	0.628	<0.05
Milk (g)	−0.557	<0.05
Oil (g)	0.598	<0.05
Salt (g)	0.598	<0.05
Fruits (g)	0.586	<0.05

**Table 10:** Spearman correlation analysis specific ingredients and blockage status.

Specific Ingredient	Spearman Correlation Coefficient (*ρ*(rho))	*p* value
Rice (g)	0.660	<0.05
Rice Flour (g)	−0.350	<0.05
Sweet Potato (g)	0.399	<0.05
Carrots (g)	0.121	≥0.05
Cabbage (g)	−0.129	≥0.05
Celery (g)	0.110	≥0.05
Pork (g)	−0.027	≥0.05
Beef (g)	0.153	≥0.05
Fish (g)	0.119	≥0.05
Milk (g)	−0.557	<0.05
Oil (g)	0.598	<0.05
Salt (g)	0.598	<0.05
Apple (g)	0.586	<0.05

### 3.6. Multiple regression and random forest model results

The results of the multiple regression model revealed that none of the nutritional components (energy, protein, fat, carbohydrates, or dietary fiber) had a significant effect on blockage status (*p* > 0.05). However, the feature importance analysis from the random forest model revealed that carbohydrates (33.33%) and dietary fiber (23.01%) were the most important features for predicting blockage status, followed by fat (22.60%) and protein (21.06%). The AUC value of this model was 0.91, indicating a strong ability to predict blockage status (Tables 11–13).

**Table 11:** Multiple regression analysis results.

Variable	Regression Coefficient	Standard Error	Z value	*p* value
Constant	−5.646	1.871	−3.017	<0.05
Energy (kcal)	0.007	0.004	1.845	>0.05
Protein (g)	−0.015	0.021	−0.714	>0.05
Fat (g)	−0.030	0.067	−0.449	>0.05
Carbohydrates (g)	−0.005	0.015	−0.312	>0.05
Dietary Fiber (g)	−0.034	0.099	−0.343	>0.05

**Table 12:** Random forest model feature importance analysis.

Feature	Importance	*p* value
Carbohydrates (g)	0.333	<0.05
Dietary Fiber (g)	0.230	<0.05
Fat (g)	0.226	<0.05
Protein (g)	0.211	<0.05

**Table 13:** Random forest model evaluation results.

Evaluation Metric	Value
Accuracy	0.805
AUC	0.907
Precision	0.833
Recall	0.75

## 4. Discussion

### 4.1. Relationships among nutritional composition, maximum injection force, and blockage status

This study provides important experimental evidence for optimizing the design of nasogastric pureed diets by analyzing the impact of different nutritional components on the maximum injection force and blockage status, with the aim of improving passability and ensuring the nutritional needs of patients. The study revealed significant positive correlations between all nutritional components (protein, fat, carbohydrate, and dietary fiber) and the maximum injection force, with the strongest correlation observed between carbohydrate content and the maximum injection force. This finding indicates that as the carbohydrate content increases, the maximum injection force also increases, suggesting that carbohydrates may significantly increase the viscosity of the pureed diet, thus affecting its passability through the nasogastric tube [11]. Multiple regression analysis further confirmed the significant impact of carbohydrates on the maximum injection force, whereas the effects of protein, fat, and dietary fiber were not significant. These results provide important guidance for formulating nasogastric pureed diets, indicating that careful control of carbohydrate content is necessary to reduce the injection force and blockage risk.

The analysis of blockage status revealed that energy, protein, fat, carbohydrate, and dietary fiber contents were positively correlated with blockage status, with significantly higher levels of these nutrients in the blocked group than in the nonblocked group. These findings suggest that excessively high nutritional density may increase the viscosity of the pureed diet, thereby increasing the resistance in the tube and increasing the risk of blockage. In particular, the content of carbohydrates and dietary fiber had a substantial effect on blockage status, indicating that these components should be carefully controlled in the formulation to reduce the likelihood of blockage.

### 4.2. Impact of ingredient selection on the injection force and blockage risk

Different ingredients significantly affect the maximum injection force and blockage risk of pureed diets. In the analysis of ingredient categories, cereals and meats presented greater correlations, which may be related to the physical properties and nutritional composition of these ingredients. Owing to their relatively high viscosity and hardness, cereals and meats tend to increase the viscosity of the pureed diet, thereby increasing the injection force demand. These findings also suggest that cereals and meats may significantly affect the textural properties of nasogastric diets, making them more prone to blockage in the nasogastric tube.

In the analysis of specific ingredients, the Kruskal‒Wallis statistic for beef was greater than that for pork and fish, indicating that beef had the greatest effect on the injection force. This may be due to the high myosin content in beef, its strong gelation ability, the structural stability of high-melting-point fats (saturated fats), the water-retention effect of abundant collagen, and the protection of the gel network by low protease activity [12]. Similarly, celery had a greater Kruskal‒Wallis statistic than carrots and cabbage did, possibly due to its higher fiber content, which increased the viscosity of the pureed diet, thereby increasing the injection force. The Kruskal‒Wallis statistic for rice was much greater than that for rice flour, as rice flour is a powdery substance derived from rice through grinding and dehydration. During the grinding process, the rice structure is disrupted, and the grains decrease in size, increasing the surface area. The starch in rice gradually gelatinizes during cooking, and the expanded starch granules form a network structure, increasing the viscosity and cohesion of cooked rice. In contrast, rice flour, owing to its smaller and more uniform particles, absorbs water quickly and gelatinizes but does not form such a stable network, resulting in a lower viscosity [13]. Rice flour and milk were significantly negatively correlated with the maximum injection force and blockage risk, possibly due to the better flowability of these ingredients, which reduced the overall viscosity of the pureed diet and thus lowered the injection force demand and blockage risk. This suggests that careful consideration should be given to the ratio of these ingredients in diet formulations, and reducing the use of high-fiber and high-hardness ingredients such as rice, sweet potatoes, beef, and celery while opting for softer, more flowable ingredients such as rice flour, fish, cabbage, and milk is recommended. This approach can optimize tube feeding procedures and improve patient comfort while maintaining nutritional balance.

Moreover, although the impact of oil and salt on the injection force was minimal, their use should still be balanced to ensure that they increase lubrication without significantly increasing viscosity.

### 4.3. Recommendations for optimizing pureed diet formulations

On the basis of the results of this study, the following recommendations can be made to optimize pureed diet formulations. First, while ensuring nutritional intake, ingredients that provide sufficient energy without significantly increasing viscosity, such as rice flour and milk, should be selected. Second, the amount of cereals and meats can be reduced or their preparation methods adjusted (e.g., increasing cooking time or grinding degree) to reduce their impact on the injection force. Rice flour can be used as a substitute for rice in cereal components. For vegetables, those with moderate fiber content and ease of pureing should be prioritized to minimize their negative impact on the viscosity of the pureed diet. In terms of energy density control, the proportion of low-melting-point fats (e.g., oleic acid) can be increased to increase the energy content rather than merely increasing carbohydrates and protein because low-melting-point fats are more likely to soften or liquefy during blending, making them less likely to form “protein‒fat complexes” and thus having a relatively minor effect on viscosity [14,15]. Additionally, commercial thickeners could be added to control the texture of the pureed diet, making it suitable for patients with swallowing difficulties while minimizing the risk of blockage [16]. The feature importance analysis from the random forest model also supports this conclusion, showing that carbohydrates and dietary fiber are crucial predictors of blockage status. Therefore, future formulation development should focus on controlling the levels of these components to optimize the balance between nutritional intake and passability.

To reduce the risk of nasogastric tube blockage caused by high-risk ingredients such as dietary fiber and meats, this study suggests increasing the viscosity of foods by using natural thickeners such as soluble dietary fiber and agar, ensuring that foods pass through the nasogastric tube smoothly while retaining their nutritional value. Furthermore, future research could explore how to retain the appearance and flavor of traditional family meals while optimizing viscosity, in order to meet the cultural and social needs of patients who wish to eat similar foods to those of their family.

This study was primarily based on a simulated nasogastric feeding process under laboratory conditions, which may differ from actual clinical feeding situations. Furthermore, this study analyzed only a few typical ingredient combinations. Future research can expand the scope to include a broader range of ingredients and different cooking methods to further validate the generalizability of the results. Future studies could focus on developing new thickeners or additives to improve the texture characteristics of pureed diets while maintaining high energy density. Additionally, individual patient differences (such as age, disease type, and swallowing function) could be incorporated to design personalized pureed diet formulations to better meet the nutritional needs and swallowing safety of different patients [17,18].

### 4.4. Risk level classification and recommendations for homemade pureed diets

On the basis of the experimental data, a five-dimensional risk level classification and intervention plan were strictly calculated. All thresholds were determined through mean difference analysis (Δ%) and effect size (Cohen’s d). The classification criteria for the risk levels are as follows (Table 14):

**Table 14:** Criteria for risk stratification of pureed diet usage.

Parameter	Blocked Group Mean ± SD	Nonblocked Group Mean ± SD	Δ% (Mean Difference)	Cohen’s d	Effect Size
Energy (kcal/d)	1753.5 ± 319.30	1218.8 ± 345.89	+43.9%	1.62	Large Effect
Protein (g/d)	90.2 ± 23.61	60.9 ± 22.11	+48.1%	1.28	Large Effect
Fat (g/d)	51.8 ± 6.29	43.0 ± 7.54	+20.5%	1.24	Large Effect
Carbohydrates (g/d)	269.7 ± 60.41	179.86 ± 55.30	+49.9%	1.52	Large Effect
Dietary Fiber (g/d)	15.9 ± 4.78	10.7 ± 3.87	+48.6%	1.18	Large Effect

Note: Cohen’s d > 0.8 is considered a “large effect,” indicating a significant clinical difference.

All thresholds are based on the mean of the nonblocked group + 1 SD (upper limit for low risk) and the mean of the blocked group – 1 SD (lower limit for high risk). The middle-risk range is between these two values, and the following risk level classifications and intervention recommendations are given(Table 15):

**Table 15:** Practical recommendations for the use of pureed diets across risk levels.

Risk Level	Energy (kcal/d)	Protein (g/d)	Fat (g/d)	Carbohydrates (g/d)	Dietary Fiber (g/d)	Intervention Plan
Low Risk	≤1400	≤70	≤45	≤200	≤12	Homemade Diet • Use rice flour instead of rice • Avoid high-fiber vegetables (e.g., celery)
	(1218.8 + 345.89 ≈ 1565, conservative value)	(60.9 + 22.11 ≈ 83, conservative value)	(43.0 + 7.54 ≈ 50.5, conservative value)	(179.86 + 55.30 ≈ 235, conservative value)	(10.7 + 3.8 ≈ 14.6, conservative value)	
Moderate Risk	1401-1600	71-85	46-55	201-240	13-15	FSMP Mixed Feeding
	(1753.5 - 319.30 ≈ 1434, rounded value)	(90.2-23.61 ≈ 66.6, adjusted to clinical practical value)	(51.8-6.29 ≈ 45.5, adjusted to clinical practical value)	(269.7- 60.41≈ 209.3, adjusted to clinical practical value)	(15.9- 4.78≈ 11.1, adjusted to clinical practical value)	
High Risk	≥1601	≥86	≥56	≥241	≥16	FSMP Full Replacement
	(1753.5 - 319.30 ≈ 1434, rounded value + 10%)	(90.2 - 23.61 ≈ 66.6, adjusted to clinical practical value + 30%)	(51.8 - 6.29 ≈ 45.5, adjusted to clinical practical value + 23%)	(269.7- 60.41 ≈ 209.3, adjusted to clinical practical value + 15%)	(15.9- 4.78 ≈ 11.1, adjusted to clinical practical value + 44%)	

These recommendations are designed to ensure a balance between adequate nutrition and minimizing blockage risk. The low-risk category recommends homemade diets, where rice flour is used instead of rice, and high-fiber vegetables such as celery are avoided. The moderate-risk category suggests a mixed feeding approach using FSMP, whereas the high-risk category advises complete FSMP replacement for feeding. This classification system provides a scientifically supported framework for managing the risk of blockage in nasogastric tube feeding while maintaining nutritional adequacy.

### 4.5. Practical implementation in clinical and home-care settings

While this study provides valuable insights into optimizing nasogastric pureed diets, translating these findings into practical clinical guidelines is crucial for improving patient care. In real-world clinical or home-care settings, several factors must be considered, including individual patient differences and variability in diet preparation. These factors can significantly affect the effectiveness of diet formulations and the risk of nasogastric tube blockage.

Individual patient characteristics, such as age, comorbidities, and the severity of dysphagia, can influence nutritional needs and the ability to safely consume pureed diets. For instance, patients with more severe dysphagia may require softer or more fluid diets, while those with milder conditions may tolerate slightly higher viscosities. Therefore, personalized approaches are essential in adjusting the formulation of nasogastric pureed diets to meet both the nutritional needs and swallowing safety of each patient.

Furthermore, real-world challenges in diet preparation can affect the consistency and nutritional balance of nasogastric pureed diets. Variations in ingredient availability, cooking methods, and home-care environments may lead to inconsistencies in the texture and viscosity of the diets. To address these challenges, healthcare providers should consider flexible strategies for preparing diets that maintain both nutritional adequacy and passability through the nasogastric tube. This may include substituting ingredients that increase viscosity (such as certain vegetables and meats) with alternatives that are easier to puree and have a lower risk of tube blockage, such as rice flour or fish.

In practice, diet adjustments should focus on ensuring that nutrient-dense foods, such as rice flour and low-fiber vegetables, are included in the formulation without excessively increasing viscosity. Additionally, strategies for overcoming common diet preparation challenges, such as improving food texture consistency and reducing high-fiber ingredients, should be incorporated into routine clinical practice to minimize the risk of blockage.

By considering these individual and environmental factors, healthcare providers can develop more tailored dietary interventions that balance the need for adequate nutrition with the prevention of feeding tube complications. This approach will ultimately improve the safety and quality of life for patients with dysphagia.

## 5. Conclusion

This study systematically analyzed the impact of nutritional composition and ingredient properties on the passability of nasogastric pureed diets and the risk of tube blockage, providing key scientific evidence for optimizing dietary formulations for patients with dysphagia. The findings indicate that energy density significantly affects the maximum injection force required for pureed diets, particularly at high energy levels (e.g., 2100 kcal), where the force demand increases substantially. This suggests that in formulation design, a balance must be achieved between high energy demands and tube passability to reduce nasogastric resistance and prevent blockage risk.

The study identified carbohydrates and dietary fiber as the primary driving factors for injection force and blockage risk, with their contents showing a significant positive correlation with the maximum injection force requirements (*p* < 0.01). Furthermore, multiple regression analysis confirmed the independent predictive value of carbohydrates (β = 0.67). With respect to ingredient selection, high-hardness and high-viscosity cereals (e.g., rice) and meats (e.g., beef) significantly increased the injection force requirements, whereas rice flour, milk, and low-fiber vegetables (e.g., cabbage) improved the flowability and reduced the blockage risk. On the basis of these findings, this study proposes an optimization strategy of substituting rice with rice flour and prioritizing fish over red meat, which can maintain the nutritional supply while reducing feeding resistance in nasogastric tube use.

By integrating five key parameters—energy, protein, fat, carbohydrate, and dietary fiber—this study developed the first nasogastric blockage risk prediction model (AUC = 0.91) and established a classification system with low, moderate, and high-risk levels along with corresponding intervention plans. Specifically, low-risk formulations (energy≤1400 kcal/d, carbohydrates≤200 g/d, protein≤70 g/d) can be homemade, whereas high-risk formulations (energy≥1601 kcal/d, carbohydrates≥241 g/d, protein≥86 g/d) require full replacement with FSMP (Food for Special Medical Purposes). This threshold-based framework provides a quantitative tool for clinical nutrition management, facilitating personalized dietary interventions for patients.

Furthermore, although the 43.1 N threshold was derived through a physical conversion from the pressure limits of commercial enteral nutrition pumps, our findings support its practical relevance within the study context. Formulations exceeding this threshold exhibited significantly higher blockage rates (*p* < 0.05), and this cutoff aligned with steep increases in carbohydrate and dietary fiber contents—two nutritional factors strongly associated with viscosity and resistance. In addition, our random forest model identified carbohydrate (importance = 33.3%) and dietary fiber (importance = 23.0%) as the most influential predictors of blockage risk, with the model achieving a strong discriminative performance (AUC = 0.91). These results suggest that the 43.1 N cutoff is both mechanistically plausible and statistically effective in identifying high-risk formulations.We acknowledge that the current threshold was validated under controlled laboratory conditions, and its direct applicability to clinical nasogastric feeding scenarios remains to be fully established. In future work, we plan to collaborate with clinical nutrition and enteral feeding teams to evaluate the performance of this threshold in real-world settings, considering influencing factors such as tube diameter, feeding posture, food temperature, and in vivo viscosity behavior. Such clinical validation will enhance the external validity and clinical translatability of our findings.

The limitations of this study include potential discrepancies between the laboratory simulation environment and actual clinical feeding scenarios, as well as the need to expand the variety and processing methods of ingredients. Future research should explore the impacts of particle size, temperature control, and processing techniques on the rheological properties of pureed diets while also developing novel thickeners to balance nutritional density and tube passability. Additionally, dynamic formulations based on swallowing function classifications and disease characteristics will be crucial for improving nasogastric feeding safety and nutritional efficacy.

The syringe-based method used in this study, while effective for controlled measurements, may not fully replicate the complex dynamics of clinical nasogastric tube feeding. It does not account for factors like food temperature, tube characteristics, or individual patient variables, which could influence feeding force in real-world conditions. Future research should consider in vivo assessments or mechanical simulations to better reflect clinical feeding dynamics and improve the relevance of the findings.

In conclusion, optimizing pureed diet formulations requires a three-pronged approach that integrates nutritional composition control, ingredient substitution, and processing improvements to minimize blockage risk while ensuring adequate nutritional intake. The findings of this study provide both theoretical and practical support for improving the quality of life and clinical safety of patients with dysphagia.

## Supporting information

S1 Dataset Correlation analysis dataset. Raw data file (Correlation Analysis.xlsx) containing correlation analyses between nutrients, ingredient categories, blockage status, and maximum injection force.
(XLSX)

S2 Dataset Descriptive dataset. Raw data file (Descriptive.xlsx) containing descriptive statistics of energy, protein, fat, carbohydrate, and dietary fiber contents across formulations.
(XLSX)
